# Perihilar FSGS lesions originate from flat parietal epithelial cells

**DOI:** 10.1007/s40620-024-01886-y

**Published:** 2024-02-01

**Authors:** Arnaldo Chia-Gil, Jürgen Floege, Eleni Stamellou, Marcus J. Moeller

**Affiliations:** 1 Division of Nephrology and Clinical Immunology, RWTH Aachen University Hospital, RWTH Aachen University, Pauwelsstr. 30, 52074 Aachen, Germany; 2 Department of Nephrology, University Hospital of Ioannina, Ioannina, Greece

**Keywords:** Focal segmental glomerulosclerosis (FSGS), Perihilar lesion, Parietal epithelial cells (PECs), Columbia classification, Secondary FSGS

## Abstract

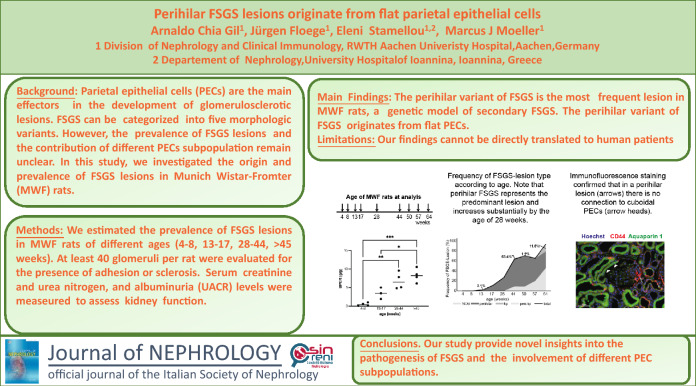

Focal segmental glomerulosclerosis (FSGS) is not a specific disease entity but rather a histological pattern of glomerular injury. It is characterized by podocyte injury, which is considered a characteristic of, and potentially a prerequisite for, the development of FSGS. However, recent evidence suggests that parietal epithelial cells are the main effectors in the development of glomerulosclerotic lesions [1]. We have recently described a novel subgroup of parietal epithelial cells, known as cuboidal parietal epithelial cells, in addition to the classical flat parietal epithelial cells. These cuboidal parietal epithelial cells represent proximal tubular epithelial cells that colonize Bowman’s capsule to varying degrees from the tubular orifice [2].

Histologically, FSGS can be categorized into five morphologic variants using the Columbia classification based on light microscopy examination [3]. These variants include collapsing, tip, perihilar, and cellular variant and FSGS not otherwise specified [4]. Although several studies from large kidney biopsy centers have validated the clinical and prognostic usefulness of the morphologic subtypes, the histologic variants alone cannot reliably differentiate between the different clinical forms of FSGS [5]. Furthermore, the factors that drive formation of the different histologic variants remain incompletely understood.

Many animal models have been developed to gain insight into the complex pathophysiology of FSGS [6]. However, the prevalence of FSGS lesions and the contribution of different parietal epithelial cell subpopulations remain unclear. Since the pathophysiology of FSGS lesions can vary between different histological patterns, understanding the cells involved in these patterns may be crucial for developing targeted therapies.

In this study, we investigated the origin and prevalence of FSGS lesions in Munich Wistar-Fromter rats. Specifically, our aim was to explore the role of parietal epithelial cells and their subpopulations in glomerulosclerotic lesion development. Munich Wistar-Fromter rats carry an unknown genetic defect that likely impairs nephron formation, resulting in reduced nephron numbers and chronic pathological hyperfiltration [7]. At ten weeks old, they develop proteinuria, and by nine months, the kidneys exhibit significant glomerulosclerosis. Focal segmental glomerulosclerosis lesions form spontaneously more frequently in male rats than in females.

Munich Wistar-Fromter rats were sacrificed in groups at the following ages: 4–8, 13–17, 28–44, > 45 weeks old (*n* = 3–4 per group). Munich Wistar-Fromter rat kidneys were perfused with NaCl 0.9% solution with a pressure of 100 mmHg before tissue embedding. Kidneys were perfused with NaCl 0.9% solution with a pressure of 100 mmHg before extraction. Perfusion was based on Bunge and Schmiedeberg and adapted for rodents [11]. Kidneys were recovered, fixed overnight in 4% formaldehyde and then embedded in paraffin. Animals were held in rooms with constant temperature and humidity, 12 h/12 h light cycles, and had ad libitum access to drinking water (ozone-treated and acidified) and standard rat chow. The German Federal Authorities (Landesamt für Natur, Umwelt, und Verbraucherschutz (LANUV) Nordrhein-Westfalen) approved all animal procedures (Az 84–02.04.2015.A517).

For light microscopy, the 4% buffered formalin-fixed kidney fragments were dehydrated and embedded in paraffin. Two-micrometer paraffin sections were stained with periodic-acid Schiff staining. At least 40 glomeruli per rat were evaluated for the presence of adhesion, sclerosis or hyalinosis. The proportion of cuboidal parietal epithelial cells in Bowman’s capsule was estimated only in glomeruli in which both the tubular and vascular pole were present.

For immunofluorescence, two-micrometer paraffin-embedded sections were stained with the following primary antibodies: CD44 *(Cell Signaling Technology, Danvers, Massachusetts, USA)*, rabbit anti-aquaporin 1 polyclonal antibody *(Abcam, Cambridge, UK)*. The following secondary antibodies were used: Alexa-Fluor-594-labeled donkey anti-mouse antibody *(Dianova, Hamburg, Germany)*, Alexa-Fluor-488-labeled chicken anti-rabbit antibody *(Invitrogen, Massachusetts, USA)*. The nuclei were stained using Hoechst 33,342 (Sigma-Aldrich, St. Louis, MO). Sections were evaluated with a Keyence BZ-9000 Microscope using BZ-II Analyzing software (Keyence Corporation, Osaka, Japan).

Levels of creatinine in serum and urine (enzymatic determination using the test kit Creatinine Plus Version 2; Roche Diagnostics) and urea nitrogen were analyzed using a Hitachi 9–17-E Autoanalyzer (Hitachi, Frankfurt am Main, Germany). Albumin level in urine was measured by a competitive two–step enzyme immunoassay using rabbit IgG to mouse albumin as first antibody (MP Biomedicals).

For assessing differences, an ANOVA or the Kruskal–Wallis test was used. Values of *p* < 0.05 were considered significant. All analyses were performed using Prism version 9.0 for Windows (GraphPad, San Diego, CA).

Animals aged without manipulation and were sacrificed for analysis at different time points, up to a maximum age of 64 weeks (Fig. 1A). Proteinuria began to increase at the age of 17 weeks and serum creatinine levels slowly increased throughout the observation period (Fig. 1B). Histological analysis revealed a steady increase in the frequency of FSGS lesions with age, starting around week 13 (2.1%) and eventually affecting almost all glomeruli (Fig. 1C). Perihilar FSGS lesions were the predominant lesion at all time points. This is noteworthy, as no other animal model or human disease is known to result in such a high frequency of perihilar lesions. Not otherwise specified FSGS lesions appeared at later time points, around week 28, and increased in frequency thereafter. Tip FSGS lesions arose at very low frequency at later time points (57 weeks). To investigate the role of cuboidal parietal epithelial cells in the formation of FSGS lesions, we assessed the presence of cuboidal parietal epithelial cells over time. In rats younger than 50 weeks, fewer than 11% of Bowman’s capsules were partially or completely colonized by cuboidal parietal epithelial cells. With higher age this colonization increased significantly (37.8 at 57 weeks) (Fig. 1D). Serial section analysis of affected glomeruli confirmed the lesion classification (Fig. 1E). De novo expression of CD44 is an established marker for parietal epithelial cell activation [8]. On confocal immunofluorescence, we found that CD44 + parietal epithelial cells in the lesions did not express aquaporin-1, which is known to be expressed only in proximal tubular epithelial cells and cuboidal parietal epithelial cells [2], thus confirming the exclusive involvement of flat parietal epithelial cells in the formation of perihilar lesions (Fig. 1F).

**Fig. 1:**
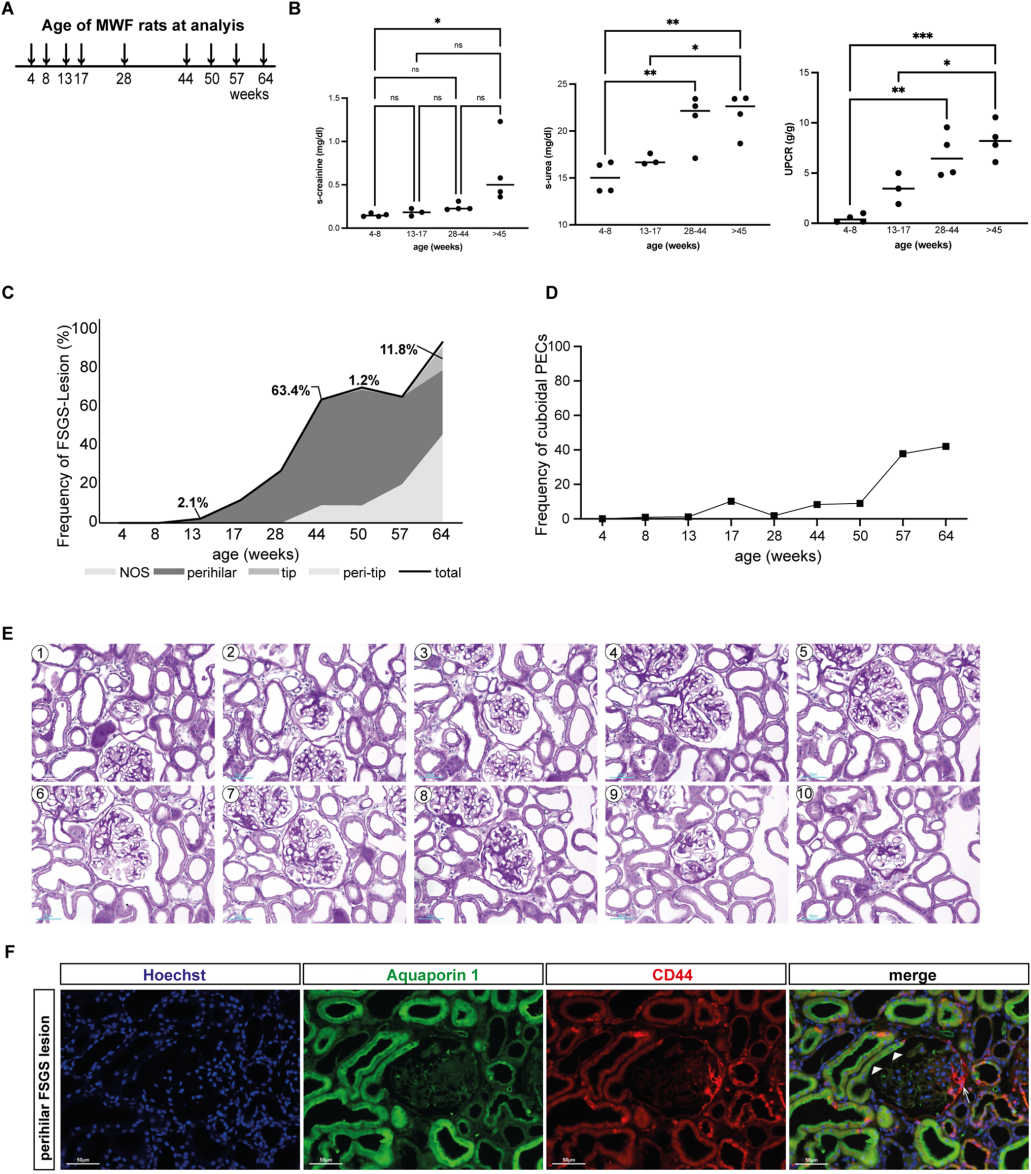
**A** Experimental set up. Rats were followed until the age of 64 weeks. **B** Renal functional parameters; serum creatinine, serum urea and urine protein/creatinine ratio (UPCR) in g/g creatinine (*n* = 4 per group). C. Frequency of FSGS-lesion type according to age. Note that perihilar FSGS represents the predominant lesion and increases substantially by the age of 28 weeks. **D** Frequency (%) of cuboidal parietal epithelial cells by age (20 glomeruli per rat,* n* = 4 per group). **E** Serial sections stained with periodic acid–Schiff (PAS). Higher magnification of picture 5 shows both tubular and vascular poles of the glomeruli. Sclerosis is depicted at the vascular pole, indicative of a perihilar lesion. Bar 50 μm. F Immunofluorescence staining of aquaporin 1 (green) and CD44 (red) confirmed that in a perihilar lesion (arrows) there is no connection to cuboidal parietal epithelial cells (arrow heads). CD44 was expressed by tubular cells in the scattered tubular cell phenotype and inflammatory cells. Additional autofluorescence staining comes from erythrocytes within the glomerular tuft

In this study, we investigated the origin and prevalence of FSGS lesions in Munich Wistar-Fromter rats. Our first major finding revealed that the perihilar variant of FSGS was the most frequent lesion in Munich Wistar-Fromter rats. These results align with previous observations in humans, where perihilar lesion formation has been associated with secondary FSGS [4, 9, 10]. Notably, no other FSGS animal models exhibit such a predominance of perihilar lesions [11]. These results underscore the importance of the Munich Wistar-Fromter rat model in replicating the characteristics of secondary FSGS observed in humans.

Moreover, our study revealed that the perihilar variant of FSGS originates from flat parietal epithelial cells. Of note, we were not able to identify a marker that specifically labels flat parietal epithelial cells, so our findings are based on the exclusion of a role of cuboidal parietal epithelial cells in the formation of perihilar lesions. Interestingly, previous findings by Kuppe et al. demontrated the role of cuboidal cells in the development of tip lesions [2]. Taken together, these studies suggest that the two main subgroups of parietal epithelial cells, i.e., flat and cuboidal parietal epithelial cells, may become activated under different experimental or pathological conditions.

It is important to acknowledge the limitations of our study. Firstly, our findings in the Munich Wistar-Fromter rat model cannot be directly translated to human patients. We have yet to identify a marker that specifically labels flat parietal epithelial cells, which limits our ability to precisely characterize their role in FSGS. We attempted to minimize sampling bias by analyzing a sufficient number of glomeruli. Nevertheless, we believe that our findings provide substantial evidence to warrant further examination of our hypothesis in human patients.

Despite these limitations, our study contributes to the existing knowledge by providing novel insights into the pathogenesis of FSGS and the involvement of different parietal epithelial cell subpopulations. The identification of perihilar lesions as the predominant variant in the Munich Wistar-Fromter rat model, along with the exclusive involvement of flat parietal epithelial cells, fills an important gap in our understanding of FSGS pathology. These findings highlight the need for further research to elucidate the underlying mechanisms and identify potential therapeutic targets.

## Data Availability

The authors confirm that the data supporting the findings of this study are available within the article. Further data are available from the corresponding author [ES] on request.
